# Occupational exposure to polycyclic aromatic hydrocarbons and cognitive impairment: Protocol of a systematic review

**DOI:** 10.1371/journal.pone.0334862

**Published:** 2025-10-17

**Authors:** Prakasit Tonchoy, Nestor Asiamah, Rufus Akintimehin, Pannawadee Singkaew

**Affiliations:** 1 School of Health and Social Care, University of Essex, Colchester, Essex, United Kingdom; 2 Department of Occupational Health and Safety, School of Public Health, University of Phayao, Phayao, Thailand; 3 Division of Interdisciplinary Research and Practice, School of Health and Social Care, University of Essex, Colchester, Essex, United Kingdom; 4 International Public Health Management Programme, University of Europe for Applied Sciences, Iserlohn, Germany; 5 Research Faculty, Berlin School of Business and Innovation, Berlin, Germany; 6 Department of Geriatrics and Gerontology, Africa Center for Epidemiology, Accra, Ghana; National Institute of Oceanography and Fisheries, EGYPT

## Abstract

Occupational exposure to polycyclic aromatic hydrocarbons (PAHs) has been associated with adverse health effects, yet evidence on the cognitive consequences of such exposure remains fragmented and has not been systematically synthesized across occupational groups. This protocol outlines a systematic review that will examine the relationship between occupational PAH exposure and cognitive impairment across eight predefined occupational categories. Relevant studies will be identified through electronic searches of major databases, including PubMed, Web of Science, Scopus, Embase, ProQuest, PsycINFO, CINAHL, the Cochrane Library, and Google Scholar. Eligible studies must assess PAH exposure in occupational settings and evaluate cognitive outcomes using validated instruments, with domain-specific neuropsychological tests prioritized as primary outcomes and global screening tools (e.g., MoCA, MMSE) included as supportive outcomes. Both observational and comparative study designs will be considered. Titles and abstracts will be screened by two independent reviewers, and full texts of potentially eligible articles will be assessed using predefined criteria. Data will be synthesized narratively, with contingency meta-analysis conducted where sufficient comparable data are available and heterogeneity is acceptable. Prespecified subgroup analyses will be undertaken by occupational category, biomarker, cognitive domain, and exposure context, with meta-regression considered if an adequate number of studies are identified. Risk of bias will be assessed using JBI tools at the study level and ROBIS at the review level, with planned sensitivity analyses. The review will consolidate current evidence on PAH-related cognitive outcomes across occupational groups, including under-studied populations such as wildland firefighters, and will inform occupational health policy, workplace screening initiatives, and future research aimed at safeguarding cognitive health in exposed populations.

## Introduction

Polycyclic aromatic hydrocarbons (PAHs) are persistent organic pollutants generated primarily through incomplete combustion of fossil fuels, biomass, and waste [1,2]. Their chemical stability and lipophilicity allow accumulation in air, water, soil, food, and living organisms [3,4]. PAHs are typically categorized into low- and high-molecular-weight (LMW and HMW) groups, with recent attention on the neurotoxic potential of LMW compounds such as pyrene and fluorene [5]. Major sources include vehicle emissions, industrial activity, biomass burning, and, increasingly, wildfires, an often-overlooked contributor [3,5]. Human exposure occurs through inhalation, dermal absorption, and ingestion of grilled and smoked foods in particular [6,7]. PAHs are associated with mutagenic, carcinogenic, and neurotoxic effects. For example, benzo[a]pyrene (BaP) is classified as a Group 1 carcinogen by the International Agency for Research on Cancer (IARC) [4,8], with mechanisms involving oxidative stress, deoxyribonucleic acid (DNA) damage, and epigenetic alterations [2,9]. Given their widespread environmental presence and multi-route exposure, PAHs remain a global health concern, particularly for their potential cognitive effects [10]. This protocol specifically focuses on PAHs because they represent one of the most prevalent and neurotoxic classes of occupational pollutants, with exposure levels often substantially higher in workers than in the general population. Their established carcinogenicity and emerging evidence of cognitive toxicity further justify prioritizing PAHs over other pollutants in this review.

A growing body of evidence links PAHs to cognitive impairment through mechanisms such as oxidative stress, neuroinflammation, and disruption of the blood–brain barrier. Experimental and population-based studies have documented mitochondrial damage, synaptic dysfunction, neuronal apoptosis, and elevated serum gamma-glutamyltransferase (an oxidative stress marker) following PAH exposure [6,9,11–13]. LMW compounds like phenanthrene and fluorene also exhibit neurotoxicity [5]. Epidemiological data support these findings: prenatal PAH exposure is associated with deficits in attention, motor function, and executive skills in children [14], while MRI studies have linked high PAH metabolite levels to cortical thinning in memory-related regions such as the hippocampus and frontal cortex [15]. In older adults, elevated urinary PAHs are associated with increased risks of mild cognitive impairment and Alzheimer’s disease [16]. Meta-analyses confirm significant associations with reduced IQ, memory loss, attention deficits, and neurodevelopmental delays [10,14].

Occupational PAH exposure is substantially higher in combustion-related professions than in the general population. A European review reported urinary 1-hydroxypyrene (1-OH-PYR) concentrations in coke production, aluminum smelting, waste incineration, and firefighting workers frequently exceeded the biomonitoring guidance value of 0.5 µg/g creatinine, sometimes by orders of magnitude [17]. In petrochemical workers, elevated OH-PAHs were associated with increased hypertension risk [18], while coke oven workers showed higher plasma p-tau231 and reduced visuospatial/executive function, suggesting early neurotoxicity [19]. Chimney sweeps exhibited urinary PAH metabolite levels up to seven times higher than controls, with strong associations to cardiovascular biomarkers such as homocysteine, cholesterol, and diastolic blood pressure [20]. Among structural firefighters, urinary 1-OHP levels doubled after fire operations, particularly among those involved in interior attacks, indicating dermal absorption despite self-contained breathing apparatus use [21]. Brain imaging studies have observed cortical and subcortical alterations in PAH-exposed firefighters [15]. Wildland firefighters also show substantial exposure, with urinary OH-PAHs increasing 6.6-fold post-suppression, accompanied by acute symptoms and immune activation [22]. Collectively, these findings underscore multiple exposure routes and health risks across a range of high-risk occupations. Several recent reviews have synthesized evidence on PAH exposure and its neurological or health outcomes [9,10,17]; however, none have specifically addressed occupational settings in relation to cognitive impairment. This protocol is therefore designed to fill this critical gap.

Despite extensive evidence linking PAHs to cognitive dysfunction, no systematic review has synthesized cognitive outcomes across occupational groups. Previous reviews have focused solely on exposure levels [17] or examined neurological disorders such as central nervous system tumors without addressing cognitive function or workplace-specific risks [10,23]. Existing primary studies tend to focus on individual occupations, such as coke oven workers [19] or firefighters [15], which limits generalizability. The innovative contribution of this protocol lies in its occupational focus and emphasis on domain-specific cognitive tests, which have not been systematically addressed in previous reviews. For instance, World Trade Center responders exhibited high rates of cognitive impairment, highlighting the broader risks of occupational exposure to combustion-derived air pollutants [24].

Wildland firefighters represent a particularly vulnerable group due to prolonged biomass smoke exposure under physically and psychologically demanding conditions. Biomonitoring studies have reported significant post-deployment increases in urinary 1-hydroxypyrene, 1-hydroxyphenanthrene, and 1-hydroxynaphthalene [25], with total OHPAH levels exceeding general population averages by more than twentyfold [26]. These exposures are largely driven by inhalation of PM₄ and volatile organic compounds that often surpass occupational exposure limits [27]. Yet, PPE practices remain inconsistent: many U.S. firefighters clean turnout gear at home and transport it in personal vehicles, contrary to NFPA standards [28], while Thai personnel face equipment shortages and extreme field conditions [29]. Face coverings like bandanas provide minimal protection [30]. Although biomonitoring is common, cognitive outcomes are rarely assessed. Canadian wildland crews report increased cognitive fatigue with longer shifts despite lacking toxicant data [31], and New York City firefighters show elevated rates of mild cognitive impairment without PAH monitoring [24]. PAH exposure has been associated with reduced cortical thickness and impaired cognition [15,19], while animal studies demonstrate that wildfire smoke disrupts neural signaling in the prefrontal cortex [32]. Few studies distinguish between cognitive outcomes from wildland and structural firefighting, and no integrated framework currently exists to evaluate exposure, biomarkers, and cognition in this high-risk group.

What remains unclear is whether existing occupational PAH biomarker studies have been systematically synthesized to elucidate the relationship between PAH exposure and cognitive impairment across diverse work environments. Variability in biomarker selection and cognitive assessment protocols, and limited data on high-risk cohorts such as wildland firefighters, further impede a comprehensive understanding.

### Objectives

The objectives of this proposed systematic review are to synthesize and compare evidence linking occupational PAH exposure to cognitive impairment across eight predefined occupational categories; to evaluate methodological variability in biomarker selection, cognitive assessment tools, and confounder control; and to identify under-studied high-risk groups (e.g., wildland firefighters) in order to inform actionable recommendations for workplace surveillance, cognitive screening, and future research priorities.

## Methods

### Review protocol and registration

This protocol follows the Preferred Reporting Items for Systematic Reviews and Meta-Analyses Protocols (PRISMA-P) 2015 guidelines [33]. A completed PRISMA-P checklist is included in S1 Table. The systematic review will be conducted in accordance with the PRISMA 2020 statement, and all subsequent stages of the review process (screening, data extraction, synthesis) will be documented in line with PRISMA requirements [34,35].

This protocol has been prospectively registered with the International Prospective Register of Systematic Reviews (PROSPERO) under the registration number CRD420251061948 [36]. Any amendments to the protocol will be updated in the PROSPERO record and clearly reported in the final review publication.

### Types of studies

Peer-reviewed articles published up to the date of the final search will be considered for inclusion regardless of publication language; non-English articles will be translated using professional services or machine-translation tools as needed to assess eligibility [37]. Eligible studies must investigate occupational exposure to polycyclic aromatic hydrocarbons (PAHs) and assess cognitive function using validated instruments. Domain-specific neuropsychological tests (e.g., memory, attention, processing speed, executive function, or other reported domains) will be considered primary measures of interest, while global screening tools such as the Montreal Cognitive Assessment (MoCA) [38] and the Mini–Mental State Examination (MMSE) [39] will be included as supportive outcomes where domain-specific measures are unavailable. Observational and comparative designs, including cross-sectional, case-control, and cohort studies, are within scope. Articles will be excluded if they are not peer-reviewed, lack full-text access, focus solely on non-occupational PAH exposure (e.g., ambient air pollution), or report exclusively on animal, in vitro, or toxicological findings without human data.

### Population and participants

Included studies must involve workers occupationally exposed to PAHs. To ensure clarity and consistency, occupational categories will be defined into eight groups: (1) wildland firefighters, (2) structural firefighters, (3) coke-oven workers, (4) aluminum smelter workers, (5) waste-incineration workers, (6) petrochemical/refinery workers, (7) chimney sweeps or combustion-service workers, and (8) asphalt/traffic-related personnel with objectively verified PAH exposure [17]. Human participants must be aged 18 years and above, and cognitive outcomes must be assessed using standardized instruments. Studies focused exclusively on the general population or children, or those lacking a clear occupational exposure context, will not be considered.

### Types of exposure

Occupational exposure to polycyclic aromatic hydrocarbons (PAHs) must be objectively verified by biomonitoring (e.g., urinary 1-hydroxypyrene, 1-hydroxyphenanthrene, or 2-hydroxyfluorene) or by area/personal air sampling within the specified occupational settings. Studies without clear occupational linkage or without objective exposure assessment will be excluded.

### Comparators

Comparative designs may involve non-exposed control groups, general population samples, or occupational groups with lower exposure levels. Comparisons across the predefined occupational categories (e.g., coke-oven workers vs. refinery workers) will also be considered. Within-group comparisons, such as wildland vs. structural firefighters, will be included when available.

### Types of outcome measures

Eligible studies must assess cognitive function using validated instruments. Domain-specific neuropsychological tests evaluating functions such as memory, attention, processing speed, executive function, or other reported domains will be considered primary outcomes for synthesis, as they provide greater sensitivity to specific cognitive deficits [40]. Global assessments (e.g., MoCA, MMSE) will be included as supportive outcomes and used where domain-specific measures are unavailable [38,41]. Where both domain-specific and global measures are reported, domain-specific results will be prioritized. Outcomes may include clinical classifications such as mild cognitive impairment (MCI) or broader indicators of long-term neurotoxic effects. Self-reported symptoms without standardized cognitive testing will not be considered.

### Search strategy

#### Electronic search.

A comprehensive search will be conducted across multiple databases to identify studies examining the relationship between occupational PAH exposure and cognitive function. The databases will include PubMed, Web of Science, Scopus, Embase, ProQuest, Google Scholar, and the Cochrane Library, as well as subject-specific databases available via EBSCOHost (e.g., MEDLINE Ultimate, APA PsycArticles, APA PsycInfo, APA PsycTests, and CINAHL Ultimate).

The search strategy will incorporate Boolean operators (OR, AND) to combine five main concept groups: (1) polycyclic aromatic hydrocarbons and their metabolites, (2) occupational exposure, (3) cognitive outcomes, (4) standardized cognitive assessments, including domain-specific neuropsychological tests (e.g., memory, attention, processing speed, executive function) and global screening tools (e.g., MoCA, MMSE), and (5) predefined occupational categories, including wildland firefighters, structural firefighters, coke-oven workers, aluminum smelter workers, waste-incineration workers, petrochemical/refinery workers, chimney sweeps or combustion-service workers, and asphalt/traffic-related personnel. Search terms will be developed using both free-text keywords and controlled vocabulary (e.g., MeSH in PubMed) for each database. Full search strings for each database are provided in S2 Table. No language filters will be applied; all records will be retrieved regardless of publication language, in line with best practice guidance to avoid language bias [42,43]. Peer-review filters will remain in place. All relevant studies published from database inception to November 2025 will be eligible for inclusion, which aligns with the planned search timeline described in the Study status and timeline section.

To assess search sensitivity, several known studies relevant to the research question will be used as reference points during pilot testing. The search strategy will be refined if these studies are not captured, thereby helping to ensure that the approach retrieves core literature before the final search is executed. This approach is consistent with recommended practices used in prior PAH-related systematic review protocols [44].

#### Non-electronic search.

As the review is limited to peer-reviewed and indexed publications, non-electronic sources (e.g., printed theses or grey literature) will not be included.

### Study selection

No language filters will be applied at the search stage; if language restrictions become necessary due to resource constraints, they will be imposed only during eligibility screening to preserve the comprehensiveness of the initial identification [43]. All retrieved records will be exported into EndNote for reference management. Duplicate entries will be automatically removed using the software’s built-in de-duplication function. A secondary manual check will be performed to identify and remove any remaining duplicates not captured by the system, particularly those with variations in author names or titles. This method follows established practices for identifying and managing duplicate records in systematic reviews [45]. After de-duplication, studies will be screened for eligibility in two stages: title and abstract screening, followed by full-text screening. The screening process will be guided by predefined inclusion and exclusion criteria, and all decisions will be documented.

#### First stage screening.

In the first stage, two independent reviewers will screen the titles and abstracts of all retrieved records to identify studies that are potentially eligible for inclusion. A structured screening checklist will be used to ensure consistency and transparency. For a study to be considered potentially eligible at this stage, the title or abstract must meet the following minimum criteria:

**Population:** The study must involve human participants aged 18 years or older who are exposed to polycyclic aromatic hydrocarbons (PAHs) in occupational settings, in line with international labour standards that set 18 as the minimum age for hazardous work [46].
**Exposure:** There must be a clear indication that PAH exposure is related to occupational activities, within one of the predefined occupational categories (e.g., wildland firefighters, structural firefighters, coke-oven workers, aluminum smelter workers, waste-incineration workers, petrochemical/refinery workers, chimney sweeps/combustion-service workers, or asphalt/traffic-related personnel).
**Outcome:** Cognitive function must be assessed using standardized or validated instruments, with domain-specific neuropsychological tests (e.g., memory, attention, processing speed, executive function, or other reported domains) considered primary measures, and global screening tools (e.g., MoCA, MMSE) included as supportive outcomes.

Studies that fail to meet any of these criteria based on the title and abstract will be excluded. The two reviewers will conduct their assessments independently. Upon completion, any discrepancies will be discussed and resolved. If disagreement persists, a third reviewer will be consulted. Inter-rater reliability between the initial two reviewers will be assessed using Cohen’s kappa statistic to evaluate the consistency of the screening process [47].

#### Second stage screening.

Full texts of all potentially eligible studies identified during the first stage will be retrieved and reviewed in detail. The same two reviewers will independently assess the full texts using the same predefined inclusion and exclusion criteria. Reasons for exclusion at this stage will be documented and categorized for reporting in the PRISMA flow diagram.

Where disagreements arise during full-text screening, the resolution process will follow that of the first stage, with initial discussion between the reviewers, followed by referral to a third reviewer if necessary. To verify screening accuracy and minimize bias, a 10% sample of included full-texts will be independently re-screened by a second reviewer, in line with PRISMA recommendations [33,35]. All steps will be conducted in accordance with PRISMA 2020 guidelines.

### Data extraction and management

Two independent reviewers will extract data from all included studies using a pre-defined and piloted data extraction sheet (S3 Table). All non-English records retrieved will be screened using machine-translation tools (e.g., Google Translate) and, where required, full texts will be professionally translated to ensure accurate data extraction and analysis [37]. The extraction form will capture bibliometric information (e.g., authors, year, country, journal), study design and setting, sample size, participant characteristics, type and method of PAH exposure assessment, and additional contextual factors including exposure context (continuous vs episodic deployments), work or deployment details (e.g., task, shift length, seasonal duration), and personal protective equipment used (e.g., respirator/N95, cloth/bandana) [22,27,30,48]. Cognitive outcome measures will be extracted, with domain-specific tests (e.g., memory, attention, processing speed, executive function, or other reported domains) prioritized as primary outcomes [40], and global screens (e.g., MoCA, MMSE) recorded as supportive outcomes [38,41]. Statistical indicators (e.g., odds ratios, confidence intervals) and any confounders controlled for in the analysis will also be recorded. These contextual variables will enable subgroup analyses and assessment of effect modification.

Prior to full data extraction, the form will be pilot-tested on a small subset of eligible studies to ensure clarity and consistency. Following the main extraction process, the completed forms will be cross-checked by a second reviewer to verify completeness and accuracy. Any discrepancies will be resolved through discussion. If consensus cannot be reached, a third reviewer will be consulted. This approach is consistent with protocols used in prior systematic reviews of occupational PAH exposure and health outcomes [44].

In cases where essential data are missing or unclear in the original publications, attempts will be made to contact the study authors via email to request clarification or additional information. If no response is received within four weeks, this will be documented, and the data will be marked as “not retrievable” in the final report.

### Data items

Data will be extracted from each included study in alignment with the review objectives. The following information will be collected:

- **Bibliographic details:** author(s), year of publication, journal name, and country of study.
- **Study characteristics**: study design, setting, sample size, participant demographics (e.g., age, gender, occupation), and inclusion/exclusion criteria.
- **Exposure details:** type of polycyclic aromatic hydrocarbons (PAHs) assessed (e.g., 1-hydroxypyrene, 2-hydroxyfluorene), method of exposure assessment (e.g., biomonitoring, air sampling), and occupational context (e.g., wildland firefighters, industrial workers). Additional variables will include exposure context (continuous vs episodic deployments), job task or role, shift length/seasonal duration, and type of personal protective equipment (e.g., respirator/N95 vs cloth/bandana). These contextual factors will be extracted to enable subgroup analyses and interpretation of potential effect modification.
- **Outcome measures:** cognitive outcomes assessed using validated instruments. Domain-specific neuropsychological tests (e.g., memory, attention, processing speed, executive function, or other reported domains) will be extracted as primary outcomes. Global assessments (e.g., MoCA, MMSE) will be extracted as supportive outcomes, and where both are available, domain-specific results will be prioritized.
- **Effect estimates:** statistical measures such as odds ratios (OR), confidence intervals (CI), p-values, or changes in cognitive test scores, including any adjustment for confounding variables.

Data extraction will be guided by the predefined extraction sheet (S3 Table), which will be pilot-tested prior to full use. Where relevant, information about funding sources, study limitations, or conflicts of interest will also be noted.

### Risk of bias assessment

Two reviewers will independently assess the methodological quality of all included studies using the Joanna Briggs Institute (JBI) critical appraisal tools, selected according to study design (e.g., cross-sectional, case-control, or cohort). These tools evaluate risk of bias across several domains, including sample selection, exposure assessment, confounding control, and appropriateness of statistical analysis. Each domain will be rated as “yes,” “no,” “unclear,” or “not applicable” following the JBI standard.

To facilitate synthesis, JBI domain ratings will be mapped to an overall judgement (low, some concerns, high) for each study. Sensitivity analyses will be conducted by excluding studies judged to have high risk of bias or inadequate control of confounders, and comparing results with and without these studies included. This process will ensure that the robustness of the findings is explicitly examined in relation to study quality. The results of risk-of-bias assessments will also directly inform the data synthesis, including planned sensitivity analyses and interpretation of pooled or narrative findings [49].

In addition, the ROBIS tool will be used to assess risk of bias at the review level (study identification and selection, data collection, and synthesis). ROBIS assessments will be reported and considered when interpreting the cumulative evidence, and will be explicitly presented in the Discussion and Limitations sections of the review [50]. Discrepancies between reviewers will be resolved through discussion, or by involving a third reviewer if consensus is not reached. No overall numerical score will be calculated; instead, domain-level assessments and mapped judgements will be documented to inform sensitivity analyses and interpretation of findings.

This approach follows established guidance on quality appraisal in systematic reviews [51] and reflects recent applications of JBI tools in environmental health research [52]. While other structured tools have also been used in PAH-related reviews, such as STROBE for reporting quality in observational studies [53] and QUADAS for diagnostic-focused evaluations [10], the JBI critical appraisal tools are chosen in this review for their structured, domain-specific applicability to various observational designs.

### Data synthesis

A narrative synthesis will be applied to integrate findings from the included studies, given the anticipated heterogeneity in study designs, exposure assessments, and cognitive outcomes. Extracted data will be organized into structured summary tables presenting occupational group, exposure type and method (e.g., biomonitoring, air sampling), PAH biomarkers measured, cognitive domains assessed (e.g., memory, attention, executive function), and effect estimates such as odds ratios or confidence intervals.

In addition to narrative synthesis, a contingency meta-analysis will be conducted if at least five studies report comparable exposure and outcome measures and heterogeneity is acceptable (I² ≤ 50%). Random-effects models (REML) will be applied [42]. Continuous outcomes will be pooled as standardized mean differences (Hedges g), while binary outcomes will be summarized as log odds ratios [54]. Prespecified subgroup analyses will examine variations by biomarker (e.g., 1-hydroxypyrene, 2-naphthol), cognitive domain (memory, attention, processing speed, executive function), occupational category, and exposure context (continuous vs episodic). Where both domain-specific and global screening measures are reported, domain-specific test results will be prioritized for synthesis and subgroup analyses. Where ≥10 studies are available, meta-regression will be explored to assess the influence of exposure dose, assessment method, and occupational category on cognitive outcomes [55]. Risk-of-bias assessments will inform both sensitivity analyses and the interpretation of pooled or narrative findings, ensuring that study quality is explicitly incorporated into the synthesis.

The synthesis will highlight areas of consistency and discrepancy between studies and identify potential methodological sources of variation. Narrative and quantitative results will be presented in accordance with PRISMA 2020 guidelines [34]. In line with recommended approaches for environmental health reviews [44] and qualitative thematic synthesis [56], data will be grouped thematically to facilitate comparison across occupational groups and cognitive domains. Tables and figures will be used where appropriate to support thematic presentation and interpretation of findings.

### Meta-bias

We will transparently report any language restrictions applied, specifying (1) the stage at which they occurred, (2) the number of studies excluded for language reasons, and (3) the rationale for any restrictions [57]. Potential duplicate publications will be identified by comparing author details, affiliations, study settings, and sample characteristics across included studies. Key study attributes recorded in the data extraction form will support this process. Selective reporting will be assessed by examining the consistency of exposure and outcome descriptions across article sections.

Formal statistical assessments of meta-bias will not be undertaken if only a narrative synthesis is possible. However, if a meta-analysis is conducted, we will evaluate potential publication bias and small-study effects using funnel plots and Egger’s regression test, and apply trim-and-fill methods as sensitivity analyses.

### Confidence in cumulative evidence

The overall confidence in the body of evidence will be assessed narratively, considering factors such as study design, methodological quality, risk-of-bias assessments (JBI at study level and ROBIS at review level), consistency of findings, and appropriateness of exposure and outcome measures. Particular attention will be given to the use of validated cognitive assessment tools, with domain-specific neuropsychological tests prioritized over global screening tools, the adequacy of PAH exposure characterization, and clarity of reporting.

Where meta-analysis is feasible, confidence in the evidence will also take into account the precision of pooled effect estimates, statistical heterogeneity (I²), and potential publication bias, as assessed through funnel plots, Egger’s regression test, and trim-and-fill methods. Summary tables and thematic groupings may be used to support interpretation and highlight areas of evidence strength or uncertainty. This approach aligns with narrative appraisal principles commonly used in qualitative reviews [56], and is supported by structured summary table strategies applicable across review types [58].

### Study status and timeline

At the time of protocol submission (June 2025), no database searches, title/abstract screening, full-text review, or data extraction have been initiated. The planned timeline for review stages is as follows:

- Database searching: October–November 2025
- Title and abstract screening: December 2025–January 2026
- Full-text screening: February–March 2026
- Data extraction and management: April–May 2026
- Data synthesis and manuscript drafting: June 2026

Because this is a protocol, no participant recruitment or new data collection has taken place to date. Any deviations from this timeline will be reported in the final publication.

### Ethics statement

This review will be based exclusively on published, peer-reviewed literature and does not involve collection of primary data from human participants. Therefore, ethical approval is not required.

## Discussion and conclusion

Occupational exposure to polycyclic aromatic hydrocarbons (PAHs) remains a pressing yet underexplored public health concern, particularly in relation to cognitive function. Although mechanistic studies [6,9] and epidemiological investigations [14,16] suggest plausible links between PAH exposure and neurocognitive decline, existing evidence is fragmented across occupational categories, exposure assessment methods, and cognitive outcomes. Prior reviews have tended to focus either on PAH exposure without addressing cognitive impairment [17], or on cognitive decline without stratifying by occupational setting [10].

Recent studies have shown associations between PAH exposure and neurodevelopmental deficits in children and depressive symptoms in adults [14], as well as reduced cortical thickness in brain regions relevant to cognition among firefighters [15]. Increased rates of cognitive impairment have also been observed in responders exposed to combustion-related air pollutants [24]. In the case of wildland firefighters, health priorities such as respiratory impacts, fatigue, and mental well-being have been identified through stakeholder consensus [59]; however, their episodic deployment patterns and variable PPE practices pose unique challenges, and cognitive effects have not yet been explicitly studied.

This systematic review will synthesize evidence linking occupational PAH exposure to cognitive impairment across eight predefined occupational categories, using narrative synthesis and contingency meta-analysis where feasible. Particular emphasis will be placed on domain-specific neuropsychological tests, with global screening tools included as supportive outcomes, and findings will be interpreted in light of study quality and risk-of-bias assessments. The review will provide three recommendations for policy, practice, and future research: first, occupational health regulators should incorporate neurocognitive endpoints into exposure limits and biomonitoring guidelines to ensure standards address both chemical and cognitive hazards; second, employers and safety agencies should implement targeted surveillance programs, such as regular cognitive screening and standardized biomarker protocols, to detect early signs of impairment in high-risk workers; and third, research funders should support longitudinal and intervention studies that evaluate dose–response relationships and assess the effectiveness of tailored controls, such as enhanced respiratory protection and structured work–rest schedules, on neurocognitive outcomes. These recommendations translate the review’s findings into concrete policy and research actions to protect brain health in PAH-exposed industries. While this review focuses on occupational PAH exposure and cognitive outcomes, it is noteworthy that broader environmental studies, such as those in materials and catalysis research [60], also address pollutant pathways and reinforce the multidisciplinary relevance of environmental health. By filling this evidence gap, the review is expected to generate novel insights into occupational cognitive risks. Its innovative scope is also anticipated to inform interdisciplinary directions, linking occupational health with environmental sustainability and broader public health perspectives. Future investigations should emphasize harmonized biomarker–cognition protocols, multi-occupational cohorts, and rigorous intervention trials to evaluate protective measures. The ultimate goal is to establish evidence-based guidelines that safeguard cognitive health in high-risk occupations and inform broader environmental and public health policy.

### Amendments

Any important amendments made after PROSPERO registration, such as changes in eligibility criteria, data synthesis methods, or outcome prioritization, will be clearly documented and reported in the final systematic review publication, along with their rationale and timing.

## Supporting information

S1 Table PRISMA-P checklist for this review protocol. (DOCX)

S2 Table Search strings and strategy for selected databases. (DOCX)

S3 Table Data extraction form template. (DOCX)
