# Supplementary material for: Occupational exposure to polycyclic aromatic hydrocarbons and cognitive impairment: Protocol of a systematic review
Source: PLoS One. 2025 Oct 17;20(10):e0334862. doi: 10.1371/journal.pone.0334862 (PMC12533870; doi:10.1371/journal.pone.0334862)
Supplement: S2 Table — (DOCX) [file pone.0334862.s002.docx]

# Appendix 2. Search strings and strategy for selected databases (PubMed, Web of Science, Scopus, EBSCOhost).

**Note:** No language restrictions will be applied to any database search. Records in all languages will be retrieved; non-English articles will be translated as needed using professional services or software.

## Search on PubMed

| **String name** | **Search terms** |
| --- | --- |
| S1 | "Polycyclic Aromatic Hydrocarbons" OR "PAHs" OR "Airborne PAHs" OR "Particulate PAHs" OR "PAH Metabolites" OR "Urinary Biomarkers" OR "Urinary Metabolites" |
| S2 | "Occupational Exposure" OR "Workplace Exposure" OR "Occupational PAH Exposure" OR "Firefighters" OR "Wildland Firefighters" OR "Structural Firefighters" OR "Coke-Oven Workers" OR "Aluminum Smelter Workers" OR "Waste-Incineration Workers" OR "Petrochemical Workers" OR "Refinery Workers" OR "Chimney Sweeps" OR "Combustion-Service Workers" OR "Asphalt Workers" OR "Traffic-Related Workers" |
| S3 | "Cognitive Impairment" OR "Neurocognitive Impairment" OR "Cognitive Decline" OR "Cognitive Dysfunction" OR "Neurocognitive Function" OR "Memory" OR "Attention" OR "Executive Function" OR "Processing Speed" |
| S4 | "Neuropsychological Test*" OR "Memory Test*" OR "Attention Test*" OR "Executive Function Test*" OR "Processing Speed Test*" OR "Montreal Cognitive Assessment" OR "MoCA" OR "Mini-Mental State Examination" OR "MMSE" |
| Final 1 | ("Polycyclic Aromatic Hydrocarbons" OR "PAHs" OR "Airborne PAHs" OR "Particulate PAHs" OR "PAH Metabolites" OR "Urinary Biomarkers" OR "Urinary Metabolites")  AND ("Occupational Exposure" OR "Workplace Exposure" OR "Occupational PAH Exposure" OR "Firefighters" OR "Wildland Firefighters" OR "Structural Firefighters" OR "Coke-Oven Workers" OR "Aluminum Smelter Workers" OR "Waste-Incineration Workers" OR "Petrochemical Workers" OR "Refinery Workers" OR "Chimney Sweeps" OR "Combustion-Service Workers" OR "Asphalt Workers" OR "Traffic-Related Workers")  AND ("Cognitive Impairment" OR "Neurocognitive Impairment" OR "Cognitive Decline" OR "Cognitive Dysfunction" OR "Neurocognitive Function" OR "Memory" OR "Attention" OR "Executive Function" OR "Processing Speed") AND ("Neuropsychological Test*" OR "Memory Test*" OR "Attention Test*" OR "Executive Function Test*" OR "Processing Speed Test*" OR "Montreal Cognitive Assessment" OR "MoCA" OR "Mini-Mental State Examination" OR "MMSE") |
| Final 2 | ('Polycyclic Aromatic Hydrocarbons'[MeSH] OR 'PAHs' OR 'Airborne PAHs' OR 'Particulate PAHs' OR 'PAH Metabolites' OR 'Urinary Biomarkers' OR 'Urinary Metabolites') AND ('Occupational Exposure'[MeSH] OR 'Workplace Exposure' OR 'Occupational PAH Exposure' OR 'Firefighters'[MeSH] OR 'Wildland Firefighters' OR 'Structural Firefighters' OR 'Coke-Oven Workers' OR 'Aluminum Smelter Workers' OR 'Waste-Incineration Workers' OR 'Petrochemical Workers' OR 'Refinery Workers' OR 'Chimney Sweeps' OR 'Combustion-Service Workers' OR 'Asphalt Workers' OR 'Traffic-Related Workers') AND ('Cognitive Dysfunction'[MeSH] OR 'Cognition Disorders'[MeSH] OR 'Cognitive Impairment' OR 'Neurocognitive Impairment' OR 'Cognitive Decline' OR 'Memory' OR 'Attention' OR 'Executive Function' OR 'Processing Speed')  AND ('Neuropsychological Tests'[MeSH] OR 'Neuropsychological Test*' OR 'Memory Test*' OR 'Attention Test*' OR 'Executive Function Test*' OR 'Processing Speed Test*' OR 'Montreal Cognitive Assessment' OR 'MoCA' OR 'Mini-Mental State Examination' OR 'MMSE') |

## Search on Web of Science

| **String name** | **Search terms** |
| --- | --- |
| S1 | "Polycyclic Aromatic Hydrocarbons" OR "PAHs" OR "Airborne PAHs" OR "Particulate PAHs" OR "PAH Metabolites" OR "Urinary Biomarkers" OR "Urinary Metabolites" |
| S2 | "Occupational Exposure" OR "Workplace Exposure" OR "Occupational PAH Exposure" OR "Firefighters" OR "Wildland Firefighters" OR "Structural Firefighters" OR "Coke-Oven Workers" OR "Aluminum Smelter Workers" OR "Waste-Incineration Workers" OR "Petrochemical Workers" OR "Refinery Workers" OR "Chimney Sweeps" OR "Combustion-Service Workers" OR "Asphalt Workers" OR "Traffic-Related Workers" |
| S3 | "Cognitive Impairment" OR "Neurocognitive Impairment" OR "Cognitive Decline" OR "Cognitive Dysfunction" OR "Neurocognitive Function" OR "Memory" OR "Attention" OR "Executive Function" OR "Processing Speed" |
| S4 | "Neuropsychological Test*" OR "Memory Test*" OR "Attention Test*" OR "Executive Function Test*" OR "Processing Speed Test*" OR "Montreal Cognitive Assessment" OR "MoCA" OR "Mini-Mental State Examination" OR "MMSE" |
| Final 1 | ALL=("Polycyclic Aromatic Hydrocarbons" OR "PAHs" OR "Airborne PAHs" OR "Particulate PAHs" OR "PAH Metabolites" OR "Urinary Biomarkers" OR "Urinary Metabolites")  AND  ALL=("Occupational Exposure" OR "Workplace Exposure" OR "Occupational PAH Exposure" OR "Firefighters" OR "Wildland Firefighters" OR "Structural Firefighters" OR "Coke-Oven Workers" OR "Aluminum Smelter Workers" OR "Waste-Incineration Workers" OR "Petrochemical Workers" OR "Refinery Workers" OR "Chimney Sweeps" OR "Combustion-Service Workers" OR "Asphalt Workers" OR "Traffic-Related Workers")  AND  ALL=("Cognitive Impairment" OR "Neurocognitive Impairment" OR "Cognitive Decline" OR "Cognitive Dysfunction" OR "Neurocognitive Function" OR "Memory" OR "Attention" OR "Executive Function" OR "Processing Speed") |
| Final 2 | ALL=("Polycyclic Aromatic Hydrocarbons" OR "PAHs" OR "Airborne PAHs" OR "Particulate PAHs" OR "PAH Metabolites" OR "Urinary Biomarkers" OR "Urinary Metabolites")  AND  ALL=("Occupational Exposure" OR "Workplace Exposure" OR "Occupational PAH Exposure" OR "Firefighters" OR "Wildland Firefighters" OR "Structural Firefighters" OR "Coke-Oven Workers" OR "Aluminum Smelter Workers" OR "Waste-Incineration Workers" OR "Petrochemical Workers" OR "Refinery Workers" OR "Chimney Sweeps" OR "Combustion-Service Workers" OR "Asphalt Workers" OR "Traffic-Related Workers")  AND  ALL=("Cognitive Impairment" OR "Neurocognitive Impairment" OR "Cognitive Decline" OR "Cognitive Dysfunction" OR "Neurocognitive Function" OR "Memory" OR "Attention" OR "Executive Function" OR "Processing Speed")  AND  ALL=("Neuropsychological Test*" OR "Memory Test*" OR "Attention Test*" OR "Executive Function Test*" OR "Processing Speed Test*" OR "Montreal Cognitive Assessment" OR "MoCA" OR "Mini-Mental State Examination" OR "MMSE") |

## Search on Scopus

| **String name** | **Search terms** |
| --- | --- |
| S1 | "Polycyclic Aromatic Hydrocarbons" OR "PAHs" OR "Airborne PAHs" OR "Particulate PAHs" OR "PAH Metabolites" OR "Urinary Biomarkers" OR "Urinary Metabolites" |
| S2 | "Occupational Exposure" OR "Workplace Exposure" OR "Occupational PAH Exposure" OR "Firefighters" OR "Wildland Firefighters" OR "Structural Firefighters" OR "Coke-Oven Workers" OR "Aluminum Smelter Workers" OR "Waste-Incineration Workers" OR "Petrochemical Workers" OR "Refinery Workers" OR "Chimney Sweeps" OR "Combustion-Service Workers" OR "Asphalt Workers" OR "Traffic-Related Workers" |
| S3 | "Cognitive Impairment" OR "Neurocognitive Impairment" OR "Cognitive Decline" OR "Cognitive Dysfunction" OR "Neurocognitive Function" OR "Memory" OR "Attention" OR "Executive Function" OR "Processing Speed" |
| S4 | "Neuropsychological Test*" OR "Memory Test*" OR "Attention Test*" OR "Executive Function Test*" OR "Processing Speed Test*" OR "Montreal Cognitive Assessment" OR "MoCA" OR "Mini-Mental State Examination" OR "MMSE" |
| Final 1 | TITLE-ABS-KEY("Polycyclic Aromatic Hydrocarbons" OR "PAHs" OR "Airborne PAHs" OR "Particulate PAHs" OR "PAH Metabolites" OR "Urinary Biomarkers" OR "Urinary Metabolites")  AND  TITLE-ABS-KEY("Occupational Exposure" OR "Workplace Exposure" OR "Occupational PAH Exposure" OR "Firefighters" OR "Wildland Firefighters" OR "Structural Firefighters" OR "Coke-Oven Workers" OR "Aluminum Smelter Workers" OR "Waste-Incineration Workers" OR "Petrochemical Workers" OR "Refinery Workers" OR "Chimney Sweeps" OR "Combustion-Service Workers" OR "Asphalt Workers" OR "Traffic-Related Workers")  AND  TITLE-ABS-KEY("Cognitive Impairment" OR "Neurocognitive Impairment" OR "Cognitive Decline" OR "Cognitive Dysfunction" OR "Neurocognitive Function" OR "Memory" OR "Attention" OR "Executive Function" OR "Processing Speed") |
| Final 2 | TITLE-ABS-KEY("Polycyclic Aromatic Hydrocarbons" OR "PAHs" OR "Airborne PAHs" OR "Particulate PAHs" OR "PAH Metabolites" OR "Urinary Biomarkers" OR "Urinary Metabolites")  AND  TITLE-ABS-KEY("Occupational Exposure" OR "Workplace Exposure" OR "Occupational PAH Exposure" OR "Firefighters" OR "Wildland Firefighters" OR "Structural Firefighters" OR "Coke-Oven Workers" OR "Aluminum Smelter Workers" OR "Waste-Incineration Workers" OR "Petrochemical Workers" OR "Refinery Workers" OR "Chimney Sweeps" OR "Combustion-Service Workers" OR "Asphalt Workers" OR "Traffic-Related Workers")  AND  TITLE-ABS-KEY("Cognitive Impairment" OR "Neurocognitive Impairment" OR "Cognitive Decline" OR "Cognitive Dysfunction" OR "Neurocognitive Function" OR "Memory" OR "Attention" OR "Executive Function" OR "Processing Speed")  AND  TITLE-ABS-KEY("Neuropsychological Test*" OR "Memory Test*" OR "Attention Test*" OR "Executive Function Test*" OR "Processing Speed Test*" OR "Montreal Cognitive Assessment" OR "MoCA" OR "Mini-Mental State Examination" OR "MMSE") |

## Search on EBSCOhost

| **String name** | **Search terms** |
| --- | --- |
| S1 | "Polycyclic Aromatic Hydrocarbons" OR "PAHs" OR "Airborne PAHs" OR "Particulate PAHs" OR "PAH Metabolites" OR "Urinary Biomarkers" OR "Urinary Metabolites" |
| S2 | "Occupational Exposure" OR "Workplace Exposure" OR "Occupational PAH Exposure" OR "Firefighters" OR "Wildland Firefighters" OR "Structural Firefighters" OR "Coke-Oven Workers" OR "Aluminum Smelter Workers" OR "Waste-Incineration Workers" OR "Petrochemical Workers" OR "Refinery Workers" OR "Chimney Sweeps" OR "Combustion-Service Workers" OR "Asphalt Workers" OR "Traffic-Related Workers" |
| S3 | "Cognitive Impairment" OR "Neurocognitive Impairment" OR "Cognitive Decline" OR "Cognitive Dysfunction" OR "Neurocognitive Function" OR "Memory" OR "Attention" OR "Executive Function" OR "Processing Speed" |
| S4 | "Neuropsychological Test*" OR "Memory Test*" OR "Attention Test*" OR "Executive Function Test*" OR "Processing Speed Test*" OR "Montreal Cognitive Assessment" OR "MoCA" OR "Mini-Mental State Examination" OR "MMSE" |
| Final 1 | S1 AND S2 AND S3 |
| Final 2 | S1 AND S2 AND S3 AND S4 |
