# Supplementary material for: Occupational exposure to polycyclic aromatic hydrocarbons and cognitive impairment: Protocol of a systematic review
Source: PLoS One. 2025 Oct 17;20(10):e0334862. doi: 10.1371/journal.pone.0334862 (PMC12533870; doi:10.1371/journal.pone.0334862)
Supplement: S3 Table — (DOCX) [file pone.0334862.s003.docx]

**Appendix 3: Data extraction form template**

| **ID** | **Bibliometric information** | | | | | | | **Exposure and Methodology** | | | | | | | **Cognitive Outcome** | | | | | | |
| --- | --- | --- | --- | --- | --- | --- | --- | --- | --- | --- | --- | --- | --- | --- | --- | --- | --- | --- | --- | --- | --- |
|  | Author(s) | Year | Country | Journal | Study Design | Study Setting | Sample Size | Occ. Group | Exposure Source | Exposure Type | Exposure Method | Exposure Context (continuous/episodic) | Task/  Deployment Details | PPE Type | Biomarkers | Sampling Type | Cognitive Domain | Assessment Tool | Outcome Type | Effect (OR, CI) | Confounders Adjusted |
| 1 |  |  |  |  |  |  |  |  |  |  |  |  |  |  |  |  |  |  |  |  |  |
| 2 |  |  |  |  |  |  |  |  |  |  |  |  |  |  |  |  |  |  |  |  |  |
| 3 |  |  |  |  |  |  |  |  |  |  |  |  |  |  |  |  |  |  |  |  |  |
| 4 |  |  |  |  |  |  |  |  |  |  |  |  |  |  |  |  |  |  |  |  |  |
| 5 |  |  |  |  |  |  |  |  |  |  |  |  |  |  |  |  |  |  |  |  |  |
| 6 |  |  |  |  |  |  |  |  |  |  |  |  |  |  |  |  |  |  |  |  |  |
| 7 |  |  |  |  |  |  |  |  |  |  |  |  |  |  |  |  |  |  |  |  |  |
| 8 |  |  |  |  |  |  |  |  |  |  |  |  |  |  |  |  |  |  |  |  |  |
| 9 |  |  |  |  |  |  |  |  |  |  |  |  |  |  |  |  |  |  |  |  |  |
| 10 |  |  |  |  |  |  |  |  |  |  |  |  |  |  |  |  |  |  |  |  |  |
| 11 |  |  |  |  |  |  |  |  |  |  |  |  |  |  |  |  |  |  |  |  |  |
| 12 |  |  |  |  |  |  |  |  |  |  |  |  |  |  |  |  |  |  |  |  |  |
| 13 |  |  |  |  |  |  |  |  |  |  |  |  |  |  |  |  |  |  |  |  |  |
| 14 |  |  |  |  |  |  |  |  |  |  |  |  |  |  |  |  |  |  |  |  |  |
| 15 |  |  |  |  |  |  |  |  |  |  |  |  |  |  |  |  |  |  |  |  |  |
| 16 |  |  |  |  |  |  |  |  |  |  |  |  |  |  |  |  |  |  |  |  |  |
| 17 |  |  |  |  |  |  |  |  |  |  |  |  |  |  |  |  |  |  |  |  |  |
| 18 |  |  |  |  |  |  |  |  |  |  |  |  |  |  |  |  |  |  |  |  |  |
| 19 |  |  |  |  |  |  |  |  |  |  |  |  |  |  |  |  |  |  |  |  |  |
| 20 |  |  |  |  |  |  |  |  |  |  |  |  |  |  |  |  |  |  |  |  |  |

*Year of study’s publication; *Country or countries where the study was undertaken; †Biomarkers include 1-hydroxypyrene, hydroxyfluorene, etc.; ‡Assessment tools include MoCA, MMSE, or other validated cognitive tests; §Exposure context coded as: 1 = continuous, 2 = episodic; PPE coded as: 1 = N95/respirator, 2 = surgical mask, 3 = cloth/bandana, 4 = none; ¶Work context may include task/role, shift length, or seasonal deployment as reported; ‖Effect estimates may include odds ratios (OR), risk ratios (RR), mean differences, or standardized mean differences (Hedges g).
